# Effects of clinoptilolite zeolite on phosphorus dynamics and yield of *Zea Mays* L. cultivated on an acid soil

**DOI:** 10.1371/journal.pone.0204401

**Published:** 2018-09-27

**Authors:** Hasbullah Nur Aainaa, Osumanu Haruna Ahmed, Nik Muhamad Ab Majid

**Affiliations:** 1 Faculty of Sustainable Agriculture, Universiti Malaysia Sabah, Sandakan Campus, Sandakan, Sabah, Malaysia; 2 Department of Crop Science, Faculty of Agriculture and Food Sciences, Universiti Putra Malaysia, Bintulu Sarawak Campus, Bintulu, Sarawak, Malaysia; 3 Institute of Tropical Agriculture and Food Security (ITAFoS), Universiti Putra Malaysia, Serdang, Selangor, Malaysia; 4 Institute of Tropical Forestry and Forest Product (INTROP), Universiti Putra Malaysia, Serdang, Selangor, Malaysia; USDA-ARS Fort Keogh Livestock and Range Research Laboratory, UNITED STATES

## Abstract

Efficient management of P fertilizers ensures good yield of crops and adequate food supply. In the acid soil of the tropics, soluble P is fixed by Al and Fe. Exploitation of the high CEC and pH of Clinoptilolite zeolite (CZ) could mitigate low soil pH and P fixation in acid soils. This study was undertaken to determine the effects of amending a weathered acid soil with CZ on: (i) soil P availability and other related soil chemical properties, and (ii) nutrient concentration, nutrient uptake, above-ground biomass, agronomic efficiency, and yield of *Zea mays* L. on a tropical acidic soil. Triple superphosphate (TSP), Egypt Rock phosphate (ERP), and Christmas Island Rock phosphate (CIRP) were used as P sources. The treatments evaluated were: (i) soil alone, (ii) 100% recommended fertilizer rate (NPK), and (iii) 75% fertilizer rate + Clinoptilolite zeolite. Selected soil chemical properties and P availability were determined before and after field trials. *Zea mays* L. above-ground biomass, nutrient concentration, nutrient uptake, agronomic efficiency, and fresh cob yield were also determined. Results revealed that the effects of treatments with and without CZ treatments on soil pH, P fractions, soil acidity, dry matter production, yield of maize, nutrient uptake, and agronomic efficiency were similar. Hence, suggesting CZ inclusion in the fertilization program of *Zea mays* L is beneficial in terms of reducing excessive or unbalanced use of chemical fertilizers due to reduction of fertilizers usage by 25%.

## Introduction

The global demand for food is increasing with the ever increasing human population. However, deprived soil fertility could impede the effort to achieve global food production and food security. Phosphorus is important in plant metabolic function, and it is one of the essential nutrients required for lucrative crops production. Phosphorus deficiency in soils does not only limit N uptake but it also leads to poor yield of crops [1,2]. Efficient uptake of P however, is related to the interactions of P with soils (sorption, desorption, and precipitation processes). Phosphorus loaded in soluble form is rapidly converted to less soluble form by adsorption and precipitation with iron and aluminium oxides thus, rendering it unavailable for crop uptake [3]. Improving P use efficiency is not only anticipated for effective crops production but it also reduces the potential of environmental pollution in surface water resulting in eutrophication and hypoxia, processes which are related to high soil P levels [4]. Non-renewable P minerals conservation is an additional long-term concern. More alarming and worrying are reports that the existing reserves of phosphate rock may last for only 50–120 years [5–7]. The responses to P scarcity due to its depletion may include increased cost, more efficient P use, P recovery, and P re-use.

Malaysia agricultural sector which contributed approximately 8.4% of national gross domestic products in 2017 [8] depends on mineral fertilizers to sustain crops productivity. The fertilizer import bills was USD$ 2.96 billion in 2008 and the bill is estimated to increase yearly [9].The use of rock phosphates is preferable in acidic conditions, because they are cheaper and their dissolution is favourable [10,11] compared with the soluble P fertilizers such as Triple superphosphate. There are many types of rock phosphate (RP) available in the market and they are: CIRP (Christmas Island Rock Phosphate), Tunisia or Gafsa (TRP), Jordan (JRP), North Carolina (NCRP), and China (CRP). These P fertilizers vary in P content. Compared to phosphate rocks, triple super phosphate (TSP) is more soluble. The choice for RP fertilizer is commonly based on market price and availability.

Phosphorus availability in acid soils can be improved by reducing P fixation in soils through chelation of metal ion oxides (Fe and Al) and competing for P adsorption site by organic acids with the use of organic amendments. The use of inorganic amendment such as zeolites to remediate P fixation is however different. Zeolites are porous crystalline, hydrated alumino-silicate minerals that form in nature as a result of a chemical reaction between volcanic lava and saline water. Zeolites have significant contribution in agriculture particularly in soil management. It is characterized as having large sorption and ion-exchange capacity, ion-exchange selectivity, properties of a molecular sieve, catalytic activity, and structural thermal stability at temperatures of up to 700–750°C. Zeolites do not easily break down over time and therefore, they remain in the soil to improve nutrients retention [12]. Besides, their hydrated amorphous silica skeleton are able to retain water and beneficial nutrients in the root zone and significantly reduce water and fertilizer costs [13]. Inclusion of clinoptilolite zeolite in agriculture also promotes N retention in soils as it improves use of NH-N and NO-N by reducing leaching losses of exchangeable cations [13–15]. Zeolites as amendment also improve nutrient uptake and nutrient use efficiency of *Zea mays* cultivated on acid soils [16–19]. Zeolites application on a sandy soil effectively ameliorated salinity stress and nutrient balance [20]. In combination with chemical fertilizers, zeolites also acted as slow-release fertilizer [21,22]. In terms of economic cost, zeolites are relatively cheap due to their abundance. Though imported from Indonesia, CZ is reasonably affordable in Malaysia and may be incorporated into fertilization programs of maize cultivation on acid soils [16]. The total production cost incurred is reduced with zeolite adoption due to elimination of liming cost and also due to reduction of fertilizers usage by 25%.

There are more than 50 natural and 150 synthetic types of zeolites. This present study used natural Clinoptilolite zeolite (CZ) which is the most widely used type not only due to abundance and low price, but also due to breadth of its physico‐chemical properties [23]. Theoretically, CZ properties, such as it being alkaline and having negative charges, can be used to improve P availability through amelioration of soil pH, reduction of soil acidity, soil exchangeable Al, and soil exchangeable Fe. This will result in less P being fixed by metal oxyhydroxides. In addition, CZ incorporation into crop fertilization programs may trigger induce-exchange dissolution mechanisms that release P through uptake of nutrients by the plant. Phosphorus release demonstrated by exchange-induced dissolution system is as follows [24]:

$$RP\;(rock\;phosphate)+NH_{4}^{+}+zeolite\to Ca-zeolite+NH_{4}^{+}+H_{2}PO_{4}^{-}$$

Isomorphous substitution of Al for Si in CZ framework provides exchange sites onto which cations are held. Plant uptake of cations from CZ leads to vacant exchange sites onto which cations are attracted. This process lowers the activity of exchangeable bases such as Ca^2+^ from soil solution thereby inducing further dissolution of rock phosphate (RP) [24–26]. Dissolution of RP is also partly related to rhizosphere acidification [27]. Crops also regulate root exudation of organic acids into soils [28,29], and it is well documented that many organic acids (such as citrate, malate, and oxalate) are the most common and effective way to mobilize poorly soluble mineral nutrients [30,31]. Their ability to reduce P precipitation [32] and even to solubilize poorly soluble phosphates [33] could be valuable in meeting crop P demands.

A prior pot experiment of an acid soil amended with P fertilizers and CZ failed to report appreciable plant response effects (above-ground biomass and nutrient recovery). However, it is worth noting that CZ incorporation in the fertilization programme is beneficial and could be used to reduce the use of N, P, and K fertilizers of *Zea mays* cultivated on acid soils [16,17,34–35]. We retested the effect of CZ amendments on maize but under field conditions and at a larger scale than the aforementioned pot experiment. Thus, field trials (2 cycles of maize cultivation) were undertaken to determine the effects of amending acidic tropical soil with CZ on: (i) dynamics of soil P availability and other related soil chemical properties, (ii) nutrient uptake, agronomic efficiency, above-ground biomass, and yield of *Zea mays* L.

## Materials and methods

### Experimental site description

A field experiment was conducted at Universiti Putra Malaysia Bintulu Sarawak Campus (latitude 03° 21.516’ N and longitude 113° 094.181’ E). The climate is humid tropical with an annual precipitation of 4042 mm, and the average monthly precipitation is 336.85 mm, and the relative humidity is 85.6%. The highest and the lowest temperatures are 32.1°C and 23.6°C, respectively. These climatic data (S1 Fig) were obtained from the Malaysian Meteorological Department report [36].

### Experimental design and treatments

The experimental design was randomized complete block design with three blocks. The treatments evaluated are summarized in Table 1.

**Table 1:** Treatments evaluated.

Treatments		Urea	P fertilizer	MOP	Clinoptilolite zeolite
		——————g plant-1 ——————			
T0	:	-	-	-	-
T1	:	4.85	4.84	2.47	-
T2	:	3.64	3.63	1.85	10.34
E1	:	4.85	7.95	2.47	-
E2	:	3.64	5.96	1.85	13.00
C1	:	4.85	7.42	2.47	-
C2	:	3.64	5.57	1.85	12.50

Note

T0 = soil alone

T1, E1, C1 = rate of fertilizer applied at 100% (recommended rate for maize cultivation), and

T2, E2,C2 = rate of fertilizer applied at 75%

Fertilizers applied consist of NPK, with differences on types of P fertilizer.

Treatments noted with ‘T’ indicates TSP, ‘E’ indicates ERP and ‘C’ indicates CIRP as source of P

Clinoptilolite zeolite as amendment was incorporated only to the treatments with 75% fertilizers rate

### Plot establishment, planting, and maintenance

Before planting, the experimental site was cleared and sprayed with herbicide (Glyphosate). Afterwards, the site was ploughed, and rotovated before 21 raised beds were constructed to accommodate seven treatments with three replicates. The size of each plot (bed) was 1.5 m (width) x 4.5 m (length) to accommodate 30 plants. A 1 m alley (distance between beds) was maintained between plots to prevent the influence of lateral water movement. Within each plot, six rows were constructed with 70 cm buffer in between. The spacing between maize plants per row was 30 cm.

The test crop used was *Zea mays* L. F1 hybrid. Five seeds were sown in each planting hole and were thinned to one at seven days after seeding. The recommended rates of the fertilizers were: urea (130 kg ha^-1^), Egypt rock phosphate (ERP) (214 kg ha^-1^), Christmas Island rock phosphate (CIRP) (200 kg ha^1^), triple superphosphate (TSP) (130 kg ha^-1^), and muriate of potash (MOP) (67 kg ha^-1^) [37]. These rates were reduced to per plant basis and were equivalent to 4.85 g urea plant^-1^, 7.95 g ERP plant^-1^, 7.42 g CIRP plant^-1^, 4.84 g TSP plant^-1^, and 2.47 g MOP plant^-1^ from the existing standard fertilizer recommendation [37]. Half the fertilizer was applied at 10 days after seeding (DAS) and the remainder at 28 DAS. This method of fertilization was used to determine the maize plants’ response to treatments (Table 1). Weeding was done manually with hoe to keep the plots free from weeds.

### Crop data collection procedures and nutrient analysis

Plants were monitored, harvested at 83 DAS and above-ground biomass was partitioned into cobs, stem, leaf, and tassel. Fresh weight of cobs were recorded using a weighing balance whereas the dry matter of stem, leaf, and tassel were determined after they were oven dried at 60°C in an oven. Each maize plant part was ground and analyzed for total N, P, and K. The dry ashing method [38] was used to extract P and K from the plant parts. The extracts were analysed for K using atomic absorption spectrophotometry (AAnalyst 800, Perkin Elmer Instruments, Norwalk, CT) whereas P was determined following the blue development [39]. Total N was determined following the micro-Kjeldahl method [40]. Nitrogen, P, and K uptake of plant parts were determined by multiplying their concentrations with their respective dry weight, whereas N, P, and K agronomic efficiency were determined using the formula shown below [41–43]:

$$Agronomic\;efficiency\;(kg\;ha^{-1})=\frac{{(Y}_{F}-Y_{0})}{F}$$

Where:

Y_F_ = Yield of fertilized (kg ha^-1^),

Y_o_ = Yield of unfertilized (kg ha^-1^), and

F = Rate of fertilizer applied (kg ha^-1^).

$$Uptake\;of\;nutrient\;(g\;plant^{-1})=Nutrient\;concerntration\;(\%)x\;dry\;weight\;(g)$$

### Determination of mineral soil, P fertilizers, and CZ properties

Before the onset of the experiment and a day before harvesting of maize fresh cobs and maize, we collected 21 soil subsamples per plot with an auger (0–20 cm depth) which were combined into one composite soil sample per plot. The 21 samples were air dried and ground to pass a 2-mm sieve for selected physicochemical characterization. Grounding is needed to reduce subsampling error as soil samples may contain clods or large aggregates. Soil samples were analyzed for pH in distilled water (at ratio of 1:2.5 soil:water) using a digital pH meter [44]. Soil particle distribution (sand, silt, and clay) was determined using the hydrometer method whereas soil bulk density was determined by the method described in the Soil Survey Staff [45]. Soil total carbon was determined using loss-on-ignition method [46]. Soil total P was extracted using aqua regia [47] whereas P concentration was measured using spectrophotometer (Lambda 25, Perkin Elmer) after blue color development [39]. Soil cation exchange capacity (CEC) was determined using the ammonium acetate method [38] whereas CEC of the CZ was determined using the CsCl method [48]. The CsCl method was used because it avoids underestimation of CEC of the zeolites as this method does not lead to entrapment of ammonium ions in the channels of zeolites. Exchangeable cations of soil, CZ (K, Ca, Mg, and Fe), and available P were extracted using double acid as extractant following the method of Mehlich No.1 [49] after which, the cations were determined using atomic absorption spectrometer (AAnalyst 800, Perkin Elmer Instruments, Norwalk, CT). Available P was determined using the Blue Method [39]. Total titratable acidity was extracted using 1 M KCl (1:10 soil/solution) after which it was determined using the colorimetric method (acid base titration) [50]. The inorganic P associated with Al, Fe, and Ca in the soil were fractionated and determined following the method described by Kuo [51]. Series of extractants were used to extract different pools of soil P. Loosely soluble-P (Sol-P) using 1 M NH_4_Cl, Aluminium bound P (Al-P) using 0.5 M NH_4_F at pH of 8.2, Iron bound P (Fe-P) using 0.10 M NaOH, and Reductant soluble P (Red-P) using 0.3 M sodium citrate and 1 M NaHCO_3._ Calcium bound P (Ca-P) was extracted using 0.25 M H_2_SO_4_, and occluded P (Occl-P) was extracted with 1 M NaOH. Extracted P fractions were measured after blue color development [39] using UV-visible Spectrometer (Lambda 25, Perkin Elmer) at 882 nm.

### Mineral soil properties

The soil physicochemical characteristics of the experimental site before planting are shown in Table 2. The soil used in this study was typical of Typic Paleudults (Bekenu series) which has an argillic horizon with fine sandy clay loam textures. This soil texture suggests possible leaching of exchangeable bases and the inherent low fertility whereas medium to coarse sub angular blocky soil structure indicates that it is generally weak and friable in nature [52]. The soil is acidic with a pH value of 4.93. Soil total Fe is 1.71 cmol(+)kg^-1^ whereas exchangeable Al in the native soil is 0.36 cmol(+)kg^-1^). This indicates that Fe is the major contributor to the acidity of this soil. The soil is also low in CEC (5.7 cmol(+)kg^-1^), total N (0.03%), total P (3.76 mg kg^-1^), and total K (2.87 cmol(+)kg^-1^). However, exchangeable K, Ca, Mg, and Na were slightly higher than the standard range reported for this type of soil [52]. Available P of this site is approximately 33% of soil total P. This relatively high level of available P is likely due to residual P from previous plantings at the site. A related study, however, reported 85 to 90% of applied P was unavailable to plants in the year of application [53].

**Table 2:** Selected soil physical-chemical properties of soil before planting of maize seeds.

Properties		Soil	
		Value obtained	Standard range*
pH (water)		4.93	4.6–4.9
Bulk Density (g cm^-3^)		1.62	Nd
CEC (cmol (+) kg^-1^)		5.7	3.86–8.46
Total N (%)		0.028	0.04–0.17
Total P (mg kg^-1^)		3.76	Nd
Available P (mg kg^-1^)		1.27	Nd
Total K (cmol (+) kg^-1^)		2.87	Nd
Total Carbon (%)		1.93	0.57–2.51
Total Fe (cmol (+) kg^-1^)		1.71	Nd
Exchangeable Al (cmol (+) kg^-1^)		0.36	Nd
Exchangeable H (cmol (+) kg^-1^)		0.52	Nd
Titratable acidity (cmol (+) kg^-1^)		0.88	Nd
Exchangeable K (cmol (+) kg^-1^)		0.22	0.05–0.19
Exchangeable Ca (cmol (+) kg^-1^)		0.49	0.01
Exchangeable Mg (cmol (+) kg^-1^)		1.15	0.07–0.21
Exchangeable Na (cmol (+) kg^-1^)		0.09	0.01
Texture		Sandy Loam	Sandy Loam
Sand (%)		62	72–76
Clay (%)		19	8–9
Silt (%)		19	16–19

*Note*: Nd = not determine

*Subjected to the soil development, standard data range by Paramananthan (2000)

The distribution of soil inorganic P (Pi) at the experimental site before planting was in the order of Fe-P > Red-P > Occl-P > Ca-P > Al-P and Sol-P (S2 Fig). Negligible Sol-P in soils indicates the need for fertilization for maize cultivation. Approximately 70% of the Pi fractions comprised active Pi (Fe-P, Al-P, and Ca-P) with 64% of it being immobilized within the crystalline and secondary minerals (associated with Fe and Al). These forms of Pi are essentially non-labile. The low Ca-P indicates low P and Ca availability in the soil, and this is because our site’s weathered soil has high levels of Fe-oxides, low pH, as well as being in advanced stage of weathering [54]. This finding is consistent with a report that Fe-P are dominant in Malaysian soils as they constitute 79% of inorganic P fractions of mineral soils [55].

### Phosphorus fertilizers and Clinoptilolite zeolite properties

The CZ (granular form) used in this study was obtained from MB Plus Sdn. Bhd. Johore, Malaysia. The selected chemical properties of TSP, ERP, CIRP, and CZ are shown in Table 3. The pH and CEC of the CZ were relatively high. The detailed information on the surface morphology and elemental composition of the CZ used in this present study can be found in one of our papers published in Geoderma [56]. The elemental content of the P fertilizers used in this present study shows that total P_2_O_5_ of TSP, CIRP, and ERP were 41%, 24%, and 27%, respectively. The Ca of the TSP, ERP, and CIRP were 4%, 47%, and 51%, respectively. The higher amount of Ca in ERP and CIRP is due to the parent materials of these fertilizers. High Ca of the apatite of the phosphates rock contributes to alkalinity of these phosphate fertilizers compared to the acidulated TSP.

**Table 3:** Selected chemical properties of phosphorus fertilizers and Clinoptilolite zeolite.

Property	TSP	ERP	CIRP	Clinoptilolite zeolite
pH (water)	2.46	7.42	7.93	8.54
CEC (cmol(+)kg^-1^)	nd	nd	nd	75.4
Total P (%)	18.09	11.96	10.62	0.01
Total P_2_O_5_ (%)	41.12	27.19	24.15	ND
Total K (%)	0.42	0.25	0.31	0.37
Total Ca (%)	4.88	47.55	51.73	0.67
Total Mg (%)	0.35	0.17	0.24	0.10
Total Fe (%)	0.38	0.61	0.52	0.11

*Note*: TSP: triple superphosphate; ERP: Egypt rock phosphate; CIRP: Christmas Island rock phosphate; nd: not determined.

### Statistical analysis

Analysis of variance (ANOVA) was used to test significant effect of treatments and followed by pairwise comparison using Tukey’s test at P = 0.05. Statistical Analysis System (SAS) version 9.2 was used for the statistical analysis.

## Results & discussion

### Selected soil chemical properties

Apart from the effort to improve P availability, understanding the transformation of added water-soluble P into various P pools and the effects of the soil properties to the P transformation process in these tropical acid soils will clarify CZ contribution on P management strategies.

Results of the selected soil chemical properties of the first planting cycle are presented in Table 4. In general, the soil pH of the two planting cycles of maize significantly increased regardless of the type of P fertilizer. The pH increased partly because of H_2_PO_4_^-^ addition caused removal of H^+^ in the soil solution of the soil with pH below 7.2 [57,58]. In the first planting cycle, the pH of the soil with 75% fertilizer and CZ (T2, C2, and E2) was lower than the recommended rate. This observation conflicts with reports that zeolites application in agriculture improves soil pH through exchange of H^+^ ions from solution with the exchangeable cations in zeolite structure [19,59]. This is related to the relatively higher Al and Fe contents in the native soil (Table 2). Because the treatments with CZ had 25% less P fertilizer, more anions were needed to saturate the sorption sites of the soil. Besides, reactivity of soil pH ranges below 5.5 is governed by Al hydrolysis. Therefore, changes of soil pH were buffered.

**Table 4:** Selected soil chemical properties at 83 days after seeding maize seeds.

	pH		Total P (ppm)		Available P (ppm)		Exchangeable (Cmol (+) kg^-1^)							
Treatments							Fe		Al		H		Ca	
	1^st^ cycle	2^nd^ cycle	1^st^ cycle	2^nd^ cycle	1^st^ cycle	2^nd^ cycle	1^st^ cycle	2^nd^ cycle	1^st^ cycle	2^nd^ cycle	1^st^ cycle	2^nd^ cycle	1^st^ cycle	2^nd^ cycle
T0	4.43^c^	4.60^b^	10.36^b^	11.67^b^	4.95^b^	4.17^c^	1.79^a^	1.10^b^	0.795	0.58	0.51^a^	0.73^a^	1.30^a^	1.25^a^
	(±0.01)	(±0.10)	(±0.46)	(±0.23)	(±0.76)	(±0.07)	(±0.02)	(±0.02)	(±0.08)	(±0.06)	(±0.01)	(±0.03)	(±0.04)	(±0.08)
T1	5.51^a^	5.21^a^	20.19^a^	21.85^a^	12.77^a^	13.62^a^	1.38^b^	1.09^b^	**	**	0.38^b^	0.28^b^	0.38^b^	0.28^b^
	(±0.04)	(±0.05)	(±0.03)	(±1.95)	(±1.19)	(±0.26)	(±0.02)	(±0.01)			(±0.06)	(±0.01)	(±0.06)	(±0.01)
T2	5.11^b^	5.46^a^	21.19^a^	19.93^a^	7.08^a^	9.36^b^	1.44^b^	1.28^a^	**	**	0.38^b^	0.29^b^	0.38^b^	0.29^b^
	(±0.03)	(±0.00)	(±1.61)	(±0.69)	(±0.65)	(±1.31)	(±0.02)	(±0.01)			(±0.04)	(±0.04)	(±0.04)	(±0.04)
T0	4.43^c^	4.60^b’^	10.36^b’^	11.67^c’^	4.95^b’^	4.17^c’^	1.79^a^	1.10^a’^	0.795	0.58	0.51^a’^	0.73^a’^	1.30^a’^	1.25^a’^
	(±0.01)	(±0.10)	(±0.46)	(±0.23)	(±0.76)	(±0.07)	(±0.02)	(±0.024)	(±0.08)	(±0.06)	(±0.01)	(±0.03)	(±0.04)	(±0.08)
E1	5.10^a^	5.55^a’^	27.82^a’^	22.53^a’^	10.82^b’^	9.02^b’^	1.63^b^	0.89^b’^	**	**	0.60^a’^	0.39^b’^	0.60^b’^	0.39^b’^
	(±0.08)	(±0.02)	(±1.48)	(±0.44)	(±0.48)	(±0.26)	(±0.01)	(±0.06)			(±0.09)	(±0.02)	(±0.09)	(±0.02)
E2	4.88^b^	5.43^a’^	30.17^a’^	30.80^b’^	18.82^a’^	11.58^a’^	1.85^a^	1.07^b’^	**	**	0.45^b’^	0.43^b’^	0.45^b’^	0.43^b’^
	(±0.12)	(±0.06)	(±1.26)	(±0.30)	(±1.37)	(±0.78)	(±0.02)	(±0.17)			(±0.07)	(±0.02)	(±0.07)	(±0.02)
T0	4.43^c^	4.60^b”^	10.36^b”^	11.67^b”^	4.95^b”^	4.17^b”^	1.79^a^	1.10^b”^	0.795	0.58	0.51^a”^	0.73^a”^	1.30^a”^	1.25^a”^
	(±0.01)	(±0.10)	(±0.46)	(±0.23)	(±0.76)	(±0.07)	(±0.02)	(±0.02)	(±0.08)	(±0.06)	(±0.01)	(±0.03)	(±0.04)	(±0.08)
C1	5.34^a^	5.39^a”^	24.01^a”^	25.75^a”^	13.05^a”^	12.03^a”^	1.52^b^	1.54^a”^	**	**	0.39^a”^	0.42^b”^	0.39^b”^	0.42^b”^
	(±0.06)	(±0.05)	(±2.05)	(±0.10)	(±1.05)	(±1.46)	(±0.31)	(±0.21)			(±0.05)	(±0.07)	(±0.05)	(±0.07)
C2	5.19^b^	5.34^a”^	26.20^a”^	22.47^a”^	11.72^a”^	13.10^a”^	1.76^a^	1.43^ab”^	**	**	0.51^a”^	0.27^b”^	0.51^b”^	0.29^b”^
	(±0.01)	(±0.04)	(±0.62)	(±1.25)	(±1.36)	(±1.22)	(±0.21)	(±0.13)			(±0.04)	(±0.02)	(±0.04)	(±0.02)

*Note*: Means with different letter indicates significant differences using Tukey’s test at *p* ≤ 0.05.

T0 = soil alone, T1, E1, C1 = 100% recommended fertilizer rate for maize cultivation, T2, E2, C2 = 75% recommended fertilizer rate.

Fertilizers applied consist of NPK, but with different types of P fertilizer.

Treatments noted with ‘T’ indicate TSP, ‘E’ indicate ERP, and ‘C’ indicate CIRP as sources of P.

Clinoptilolite zeolite as amendment was incorporated only to the treatments with 75% fertilizers rate

Irrespective of treatment, soil exchangeable Al in both planting cycles significantly decreased to negligible whereas exchangeable Fe reduction was inconsistent (Table 4). Such observation is due to the strong sorption of P that was governed by the soil pH and also reactivity of different types of P fertilizer used. Strong P sorption by Al occurred at pH of soil <5.5 whereas at the pH <4.5, P is strongly sorbed by Fe [60]. Besides, although CZ inclusion in this present study was not very effective in increasing soil pH compared to the recommended rate, the slight effect on pH might have contributed to Al reduction. Similar to our findings, Basak and Biswas [61] study on liming effect of low grade rock phosphates revealed low increase in acid soil pH. However, the reduction in available Al played significant role in improving plant growth environment in the strong acid soils. Moreover, the presence of H^+^ partially alleviates the phytotoxic effect of Al by competing with Al^3+^ at the root cell plasma membrane [62]. At higher pH values, where monomeric hydroxyl Al species predominates, the activity of H^+^ is reduced such that its competitive effects are significantly reduced. This explains the insignificant difference of H^+^ in the soil regardless of the rate and type of P fertilizer applied.

### Phosphorus availability

Relative to control, significant P availability is noted in all P fertilizer treatments regardless of the rate and type of P fertilizer applied. The addition of CZ to the treatments with 75% fertilizers rate (T2, E2, and C2) showed comparable total P and available P with the recommended rate in both the first and second planting cycles (Table 4). Inclusion of CZ in these treatments mitigated soil pH besides reducing Al, and soil acidity. Hence, resulting in lesser amount of P being fixed to the soil colloid and metal ion oxyhydroxides although lesser amounts of P fertilizers were applied. Moreover, the comparable soil total P, exchangeable P, and soil exchangeable Ca contents (Table 4) obtained was possible due to enhanced dissolution of the PRs as affected by induce-exchange mechanism of the CZ mediated by maize plant uptake. Adequate supply of moisture, protons (H^+^), and exchange site by CZ could have provided a sink for removal of reactive products such as Ca^2+^ and H_2_PO_4_^-^. Thus, suggesting the dissolution reaction from left to right to increase P availability $\left[Ca_{10}(PO_{4})F_{2}+12H^{+}\to 10Ca^{2+}+6H_{2}PO_{4}^{-}+2F^{-}+12OH^{-}\right]$.

With the exception of ERP, the use of other RPs as P source regardless of rate increased soil P availability above the critical level (10 mg kg^-1^) for maize plants [63]. The higher available P recovered in E2 (25% less fertilizer) compared with E1 suggests that it is possible to reduce ERP use in maize cultivation. The finding also indicates that co-application CZ and less soluble RP unlike highly soluble P (TSP) optimizes P fertilizers use in maize production on acid soils.

### Dynamics of phosphorus speciation

Phosphorus bioavailability relates to the speciation of P in the soil solution. Commonly, free orthophosphate is not the dominant P species in the soils solution, especially in soil rich in Al/Fe oxides. In these soils, mobile colloidal P could potentially contribute to P availability.

In the first and second planting cycles, the 70 to 80% of Pi in the soil without CZ and fertilizers (T0) were primarily three active fractions, namely Fe-P, Al-P, and Ca-P (S2 Fig). The distribution of the Pi fractions was in the order of: Fe-P > Ca-P > Occl-P > Red-P > Al-P > Sol-P of first planting cycle whereas Fe-P > Red-P > Ca-P > Occl-P > and Al-P was the order for the second planting cycle. The dominance of Fe-P pre- and post-treatment (S2 Fig) indicates that Fe controls the dynamics of P especially P sorption in agricultural soils [64–66] and served as sink for applied P [67,68]. The changes in the order of Pi fractions is due to the dynamics transformations of applied P fertilizers which are different in terms of mobility, bioavailability, and chemical behavior under different conditions [69].

**Fig 1:**
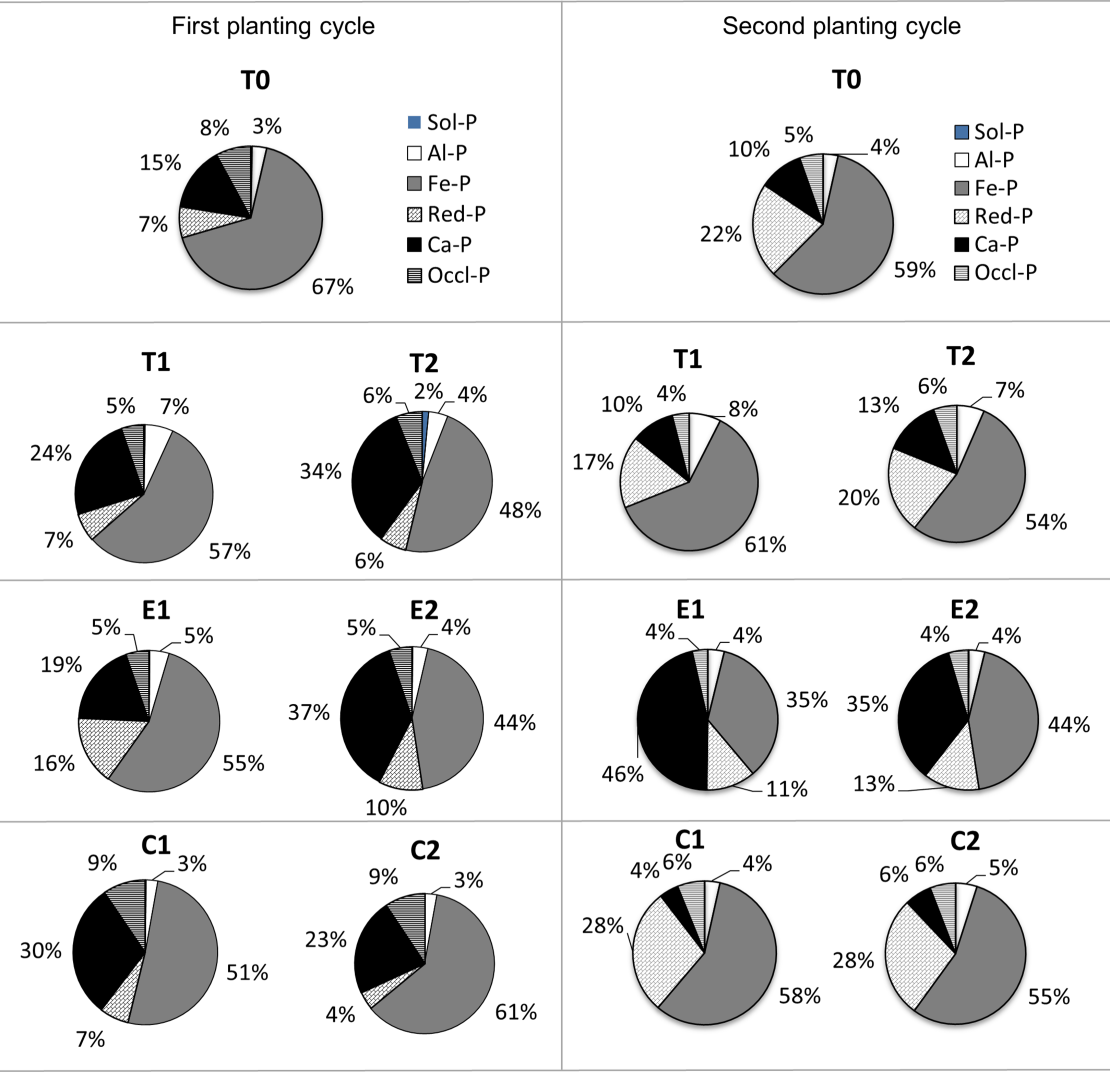
Percentage of inorganic phosphorus speciation in soil after two cycles of maize cultivation. Sol-P: loosely soluble-P, Al-P: aluminium-P, Fe-P: Iron-P, Red-P: reductant-P, Ca-P: calcium-P, and Occl-P: occluded-P.

The Sol-P levels were low regardless of fertilization and planting cycle (Fig 1). Following TSP application (highly soluble P), Sol-P was low not only due to uptake by the maize plants and leaching but also due to its conversion to less available forms following P release from P fixation. The low Sol-P of RPs was due to its slow release which was facilitated by soil chemical properties (soil pH, concentration of Ca, and P in soil solution). In addition, availability of P is not only governed by parent material and weathering stage, but also by biological processes such as plant uptake, microbial uptake, immobilization, and mineralization.

In the second planting cycle, the increase in Red-P was more pronounced regardless of the rate and type of P fertilizer applied. Saturation levels of the P in soils could have increased due to the previous cultivation. This finding corroborates those of Kolachi and Jalali [70] and Zhang and Mackenzie [71,72] who also found that this moderately labile P species also serve as P sink. Unlike in the first planting cycle, the recommended P rates (regardless of P fertilizer type) consistently reduced Red-P in the second planting cycle. The observations about Red-P are related to drainage conditions of the soils during cultivation of the maize. This is because, solubilization of Fe-P complexes in Red-P through reduction of Fe (III) minerals of a tropical soils require prolonged anaerobic conditions for P to become available [73,74].

The low content of Ca-P (Fig 1) in two planting cycles is in line with soil acidity and advanced pedogenesis, indicating significant amount of native apatite had been weathered [75]. This observation corroborates findings by Melese *et al*. [76] and Adhami *et al*., [77] but contradicted those reported for less weathered soils. However, the contrary observation of Ca-P following application of ERP treatments in this study could be due to the undissolved rock phosphate. The use of ERP consistently resulted in the highest Ca-P throughout the two planting cycles of maize compared to TSP and CIRP because of slower dissolution of ERP.

Regardless of fertilizer reduction and CZ inclusion, fixation of P in Occl-P was similar to the recommended fertilization of RP treatments (Fig 1). This suggests that P adsorption was reduced by CZ application and this is evident in the comparable P availability (Table 4) and P adsorbed such as Al-P, Fe-P, and Ca-P, (Fig 1) despite 25% less fertilizer. The similar levels of P fixed in Occl-P may also be due to competition of organic acids with phosphate ions for adsorption site produced from soil microbial activity and saturation of soil sorption sites by P added to the soil [78]. Besides, root exudation produces various organic acids (citrate, malate and oxalate) [28,29] that help to mobilize poorly soluble mineral nutrients and P [30,31]. These exudates enable reduction of P precipitation [32] and solubilization of poorly soluble phosphates [33] which could be valuable in meeting plants’ demand for P.

### Maize yields and dry matter production

**Fig 2:**
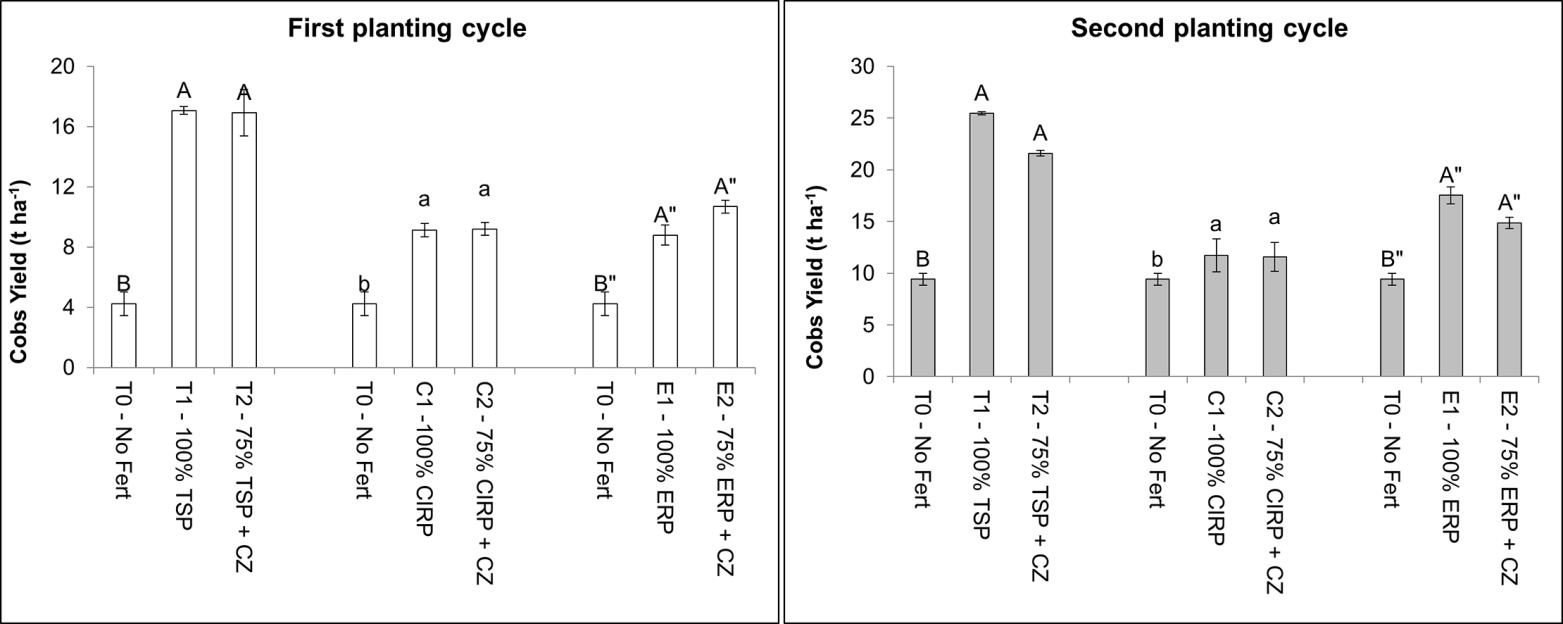
Cobs yield after two cycles of maize cultivation. Means with different letter indicates significant differences using Tukey’s test at p ≤ 0.05.

In spite of 25% reduction of fertilization in the first and second planting cycles, the treatments with CZ showed similar cobs yield compared to the existing fertilizer recommended rate (Fig 2). The maize plant dry matter production is shown in Table 5. Regardless of P fertilizer type, 75% fertilizer amended with CZ resulted in comparable dry matter production as the fertilizer recommended rate. This result is consistent with those reported in our pot studies [17,34,35] thus, indicating the beneficial effects of CZ as a slow release fertilizer. Therefore, CZ addition along with 25% reduction of fertilization in the planting cycles suggests a need for reduction in the existing fertilization especially for long term maize cultivation.

**Table 5:** *Zea mays* L. plant dry matter production in two cycles of cultivation.

Treatments	Leaf				Stem				Tassel				Total			
	1^st^ cycle		2^nd^ cycle		1^st^ cycle		2^nd^ cycle		1^st^ cycle		2^nd^ cycle		1^st^ cycle		2^nd^ cycle	
T0	8.02^b^	±0.22	13.77^b^	±1.43	18.89^b^	±0.59	36.42^b^	±2.75	1.94^b^	±0.11	3.10^b^	±0.13	28.85^b^	±1.49	53.29^b^	±4.31
T1	17.65^a^	±1.65	35.64^a^	±2.29	44.79^a^	±3.00	77.65^a^	±2.96	3.77^a^	±0.01	5.24^a^	±0.19	66.21^a^	±1.76	118.54^a^	±5.43
T2	19.90^a^	±1.61	41.29^a^	±4.67	49.32^a^	±5.19	84.86^a^	±1.61	3.83^a^	±0.07	5.05^a^	±0.43	73.04^a^	±7.37	131.21^a^	±6.71
C0	8.02^b’^	±0.22	13.77^b’^	±1.43	18.89^b’^	±0.59	36.42^b’^	±2.75	1.94^b’^	±0.11	3.10^b’^	±0.13	28.85^b’^	±1.59	53.29^b’^	±4.31
C1	13.25^a’^	±2.16	31.16^a’^	±2.70	28.37^a’^	±1.68	59.45^a’^	±3.17	2.87^a’^	±0.16	5.37^a’^	±0.36	44.49^a’^	±5.21	95.97+	±6.23
C2	11.32^a’^	±1.36	28.44^a’^	±0.09	33.45^a’^	±2.49	55.02^a’^	±0.37	2.63^a’^	±0.15	4.36^ab’^	±0.54	47.40^a’^	±6.01	87.83^a’^	±1.00
E0	8.02^b”^	±0.22	13.77^b”^	±1.43	18.89^b”^	±0.59	36.42^b”^	±2.75	1.94^b”^	±0.11	3.10^a”^	±0.13	28.85^b”^	±1.49	53.29^b”^	±4.31
E1	12.91^a”^	±1.09	22.48^a”^	±1.30	28.16^a”^	±2.16	47.83^a”^	±0.75	2.69^a”^	±0.26	3.23^a”^	±0.23	43.76^a”^	±4.80	73.55^a”^	±2.28
E2	12.14^a”^	±0.74	19.90^a”^	±0.98	27.84^a”^	±3.74	45.45^a”^	±0.55	2.87^a”^	±0.19	3.26^a”^	±0.03	42.85^a”^	±5.19	68.61^a”^	±1.55

Note: Means with different letter indicates significant differences using Tukey’s test at *p* ≤ 0.05.

T0 = soil alone, T1, E1, C1 = 100% recommended fertilizer rate for maize cultivation, T2, E2, C2 = 75% recommended fertilizer rate.

Fertilizers applied consist of NPK, but with different types of P fertilizer.

Treatments noted with ‘T’ indicate TSP, ‘E’ indicate ERP, and ‘C’ indicate CIRP as source of P.

Clinoptilolite zeolite as amendment was incorporated only to the treatments with 75% fertilizers rate

### Nutrient uptake and agronomic efficiency

**Fig 3:**
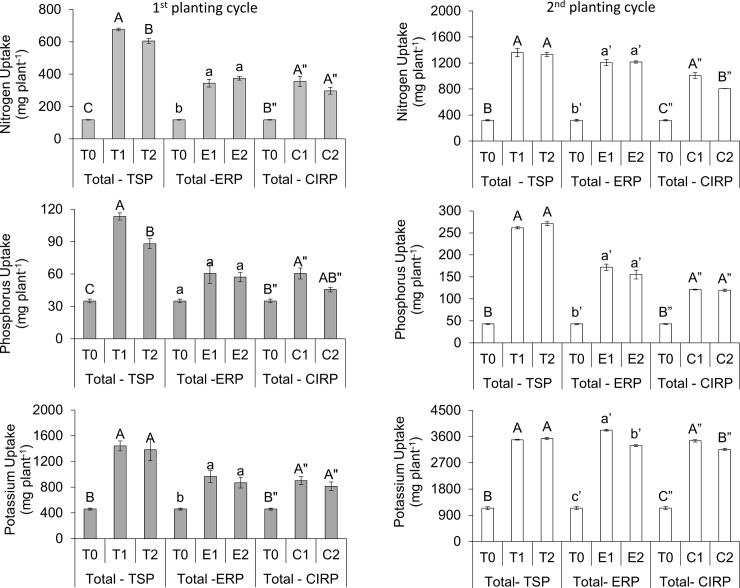
Total N, P, and K uptakes after two cycles of maize cultivation. Means with different letter indicates significant differences using Tukey’s test at p ≤ 0.05. The error bars are the ± standard error.

**Fig 4:**
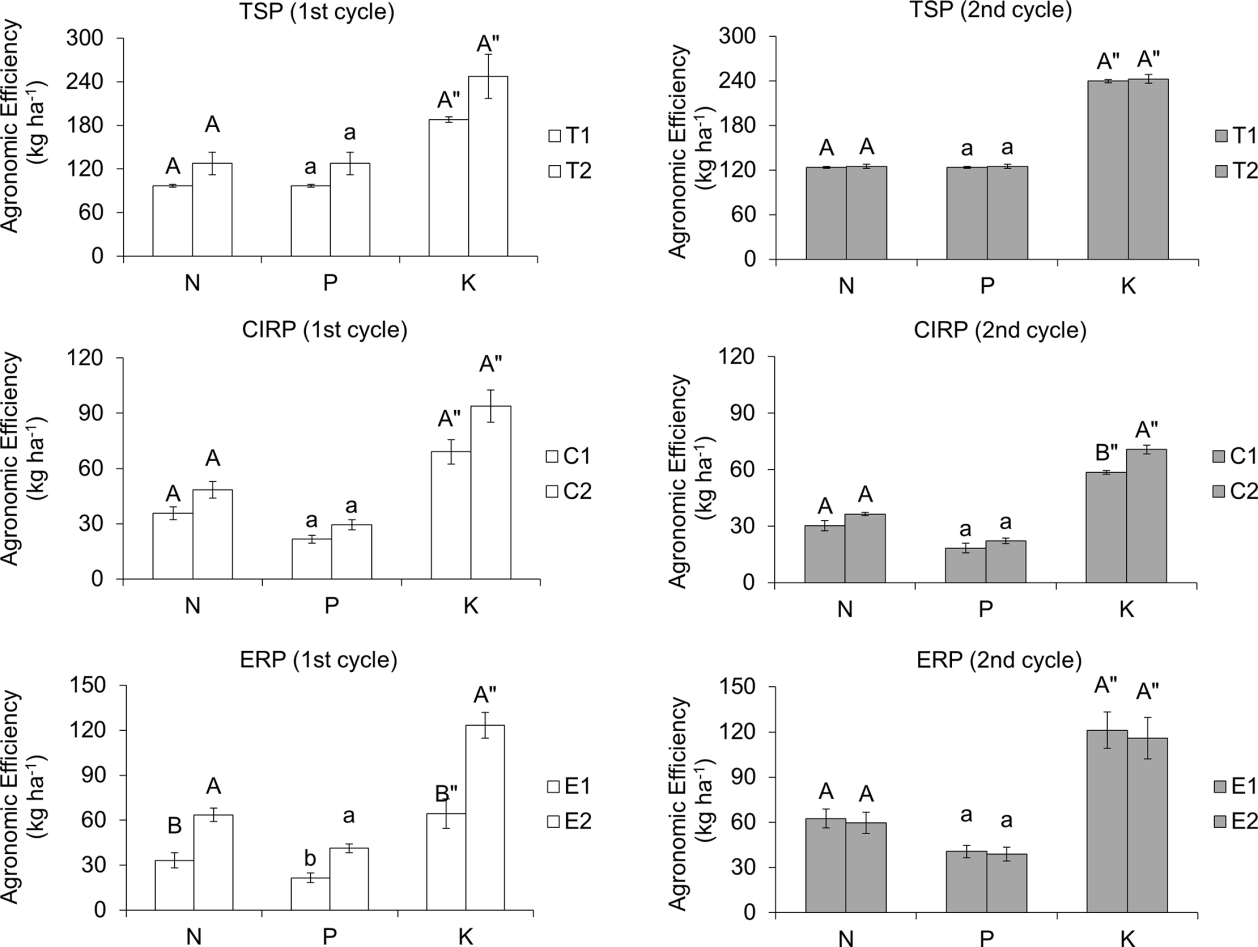
Agronomic efficiency of N, P, and K after two cycles of maize cultivation. Means with different letter indicates significant differences using Tukey’s test at p ≤ 0.05. The error bars are the ± standard error.

Uptakes of N, P, and K by maize plants of the first cycle are summarized as total uptake of respective nutrients in Fig 3. Total N and K uptake in the maize plant show that CZ with 75% fertilization and the recommended fertilization rate had similar effect on N and K uptake. This is possible because of the high CEC and affinity of CZ for NH_4_^+^ and K^+^. For NH_4_^+^ in particular, it is possible the affinity of CZ for NH_4_^+^ might have not only reduced nitrification but it also might have facilitated slow release of NH_4_^+^ to prevent it from being lost through leaching. Furthermore, the presence of CZ reduced microsite pH by inhibiting ureolytic activity of microorganisms to minimize ammonia volatilization from NH_4_^+^ [18,79–81]. Additionally, the cation selectivity of the CZ which is in the order of K^+^ > NH_4_^+^ > Na^+^ > Ca^2+^ > Mg^2+^ [82,83] relates to the aforestated observation. With the exception of ERP treatment (75% of recommended), other treatments at 75% of recommended showed similar N, P, and K agronomic efficiencies as compared to the treatments at recommended fertilization levels (1^st^ planting cycle) (Fig 4). Total P uptake of the plants of the first planting cycle shows that the use of TSP in the treatments resulted in significant reduction of P uptake for 75% fertilization amended with CZ (T2) whereas the use of RP fertilizers showed no significant difference. The higher P uptake in the TSP treatments was due to the higher solubility of the TSP compared with the RPs as the dissolution of this RPs depends on soil acidity and concentrations of Ca and P ions present in the soil solution. The agronomic efficiency of the CZ with 75% fertilization was similar to those of the recommended rates of TSP and CIRP, whereas in the ERP treatments, P agronomic efficiency significantly increased with 75% fertilization and CZ (Fig 4).

In the second planting cycle, N uptake in the treatments with lower TSP and ERP were comparable to the 100% fertilization except for CIRP (Fig 3). Phosphorus uptake was similar regardless of the rate of P (Fig 3). For the TSP treatments, K uptake was similar, whereas in the RPs treatments, K uptake was significantly lower than the recommended rate (Fig 3). The higher nutrient uptake in the second planting cycle in addition to the similar N, P, and K agronomic efficiencies (Fig 4) explain the higher cobs yield attained in the second planting cycle (Fig 2). This finding is in agreement with that of Cassman *et al*. [84] who also observed that nutrient use efficiency is greatly affected by the amount of nutrients used and by synchronization between demand and supply of the nutrients.

Although the effects of CZ inclusion in the fertilization program of *Zea mays* were not significantly different (in terms selected soil chemical properties, cobs yield, uptake and agronomic efficiency of N, P, and K), the comparable results relative to those of the recommended rate suggest the need to reduce the use of chemical fertilizers to avoid losses and environmental pollution. The similarity in the nutrient uptake and use efficiency was due to the ability of the CZ to temporarily retain the nutrients and timely release of cations. Besides, the lack of significant effect was due to the amounts of CZ and fertilizer used in this present study were lower (for economic reasons) than those reported in other studies [14,22,19,85]. Probably, in long term application, significant results could be obtained as noted in other studies where inorganic fertilizers were mixed with zeolite [17,19,21]. Inclusion of zeolite in fertilization programmes is reported to cause a phenomenal growth of microorganisms in soils, increase in nutrient intake by plants, ameliorate negative effects of soil acidity, prevention of soil erosion, increase cations storage capacity of soils, and building of precious humus complexes [86,87].

## Conclusion

By amending a weathered acid soil with CZ, the existing fertilizer use in maize cultivation can be reduced by 25% as with and without CZ treatments showed similar effects on soil pH, P availability, basic cations, soil acidity, soil exchangeable Al, dry matter production, yield of maize, nutrient uptake, and agronomic efficiency. Amending tropical mineral acid soils with CZ is a potential approach to reduce the need for chemical fertilizers in maize cultivation. For economic and environmental considerations, the use of CZ in agriculture is beneficial as it can be used to reduce the unbalanced use of N, P, and K fertilizers of *Zea mays* L. and related crops cultivated on acid soils besides minimizing environmental pollution due to excessive use of chemical fertilizers.

## Recommendation

Results from two cycles of maize cultivation may not be conclusive enough to confirm the findings. Actual remedial and conditioning effects of Clinoptilolite zeolite perhaps require more time. Therefore, several more cycles of maize cultivation at different locations and at a larger scale will be needed. Climatic factors such as temperature and rainfall distribution between planting cycles should also be taken into consideration to confirm the findings of this study.

## Supporting information

S1 Fig Initial percentages of inorganic P speciation in Typic Paleudults. Sol-P is loosely soluble P, Al-P is aluminium bound P, Fe-P is iron bound P, Ca-P is calcium bound P, Red-P is reductant P, and Occl-P is occluded P.
(TIF)

S2 Fig Mean monthly rainfall, maximum temperature, and minimum temperatures of the study area (Source:Malaysian Meteorological Department, 2014). (TIF)

S3 Fig Mechanism of P uptake and efficient use with zeolite intervention in an acid soil. (TIF)
