# Supplementary figures and images for: Effects of clinoptilolite zeolite on phosphorus dynamics and yield of Zea Mays L. cultivated on an acid soil
Source: PLoS One. 2018 Sep 27;13(9):e0204401. doi: 10.1371/journal.pone.0204401 (PMC6160028; doi:10.1371/journal.pone.0204401)

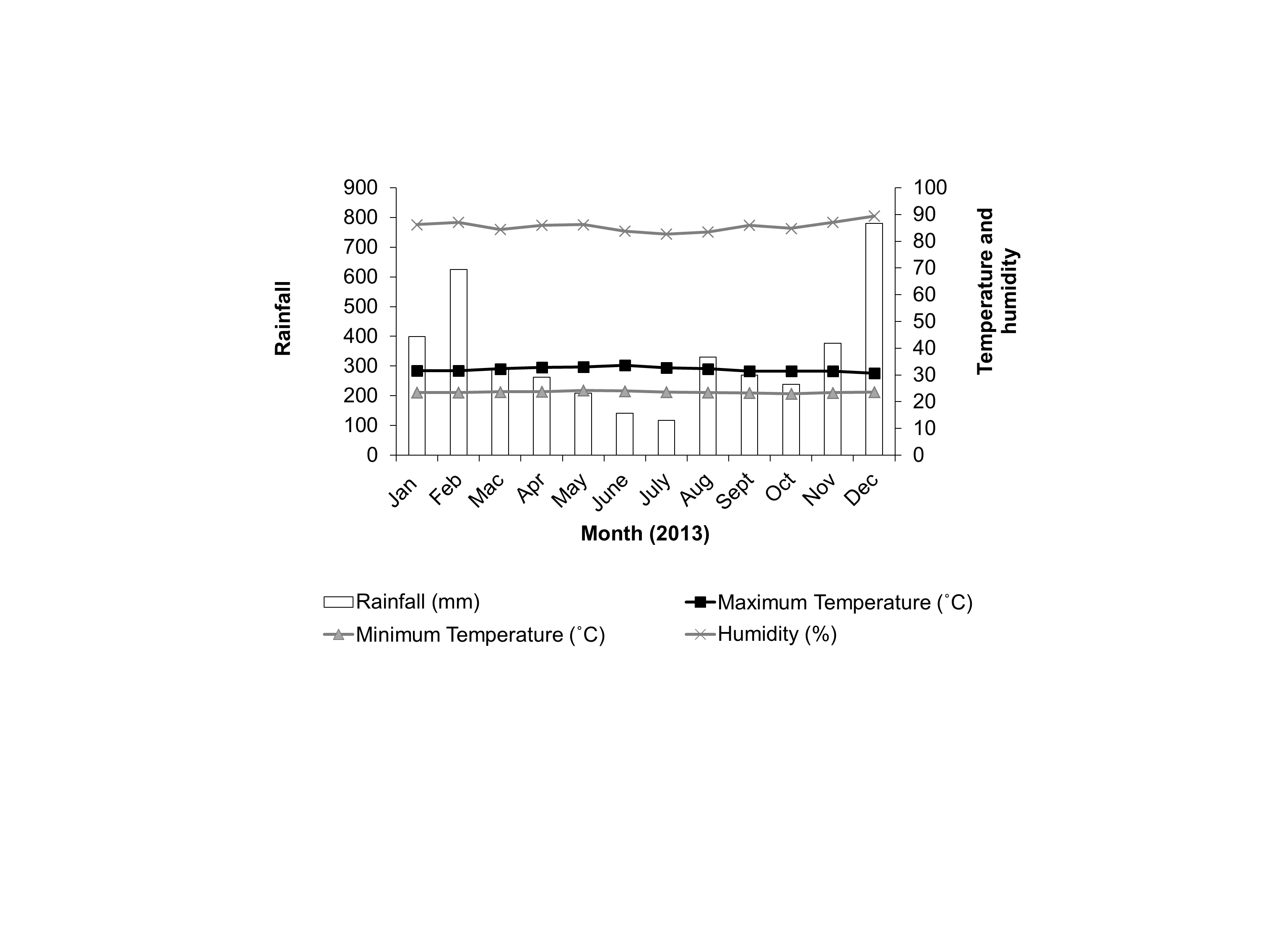

Supplement: S1 Fig — Sol-P is loosely soluble P, Al-P is aluminium bound P, Fe-P is iron bound P, Ca-P is calcium bound P, Red-P is reductant P, and Occl-P is occluded P. (TIF) [file pone.0204401.s001.TIF]

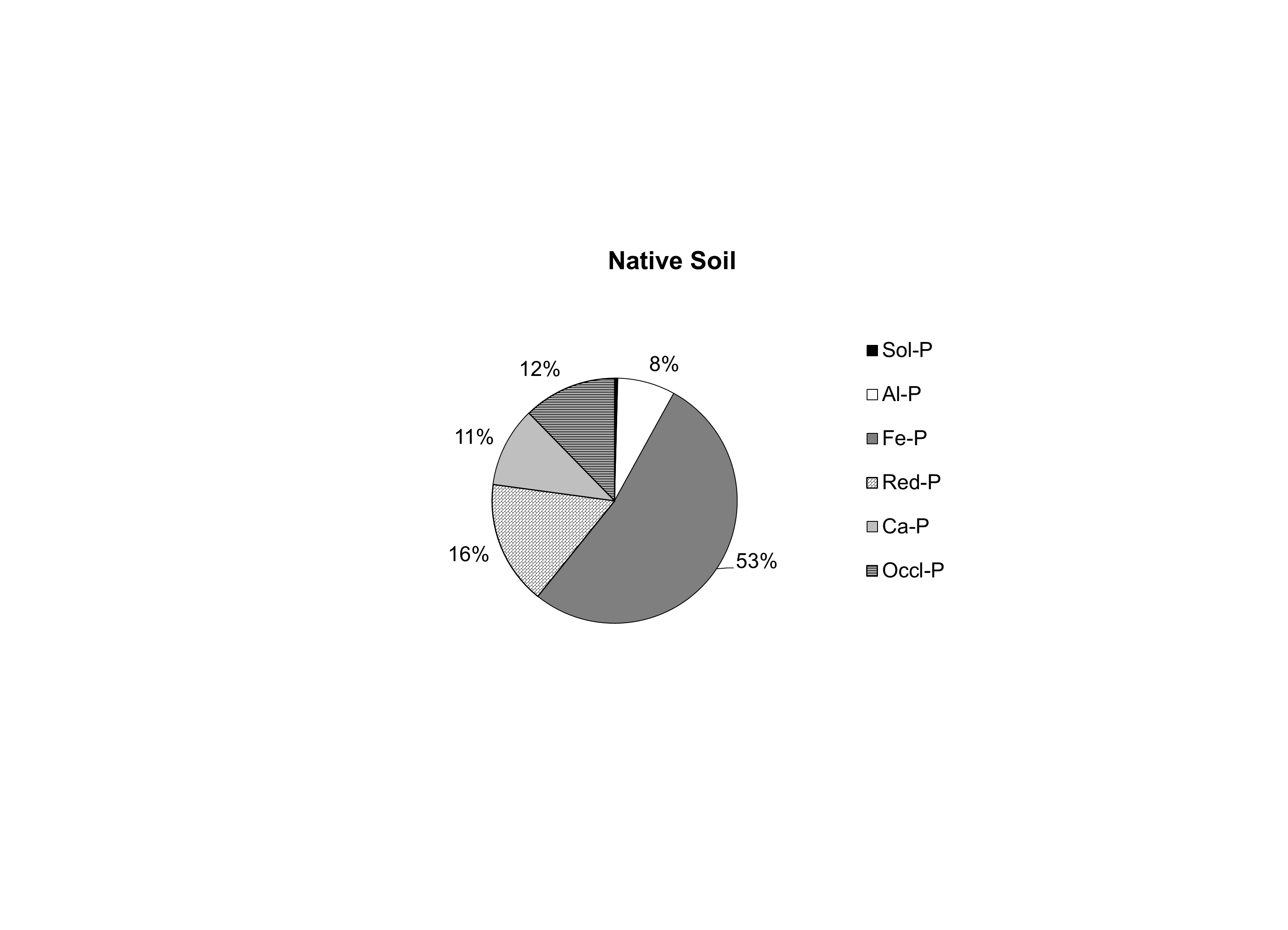

Supplement: S2 Fig — (TIF) [file pone.0204401.s002.TIF]

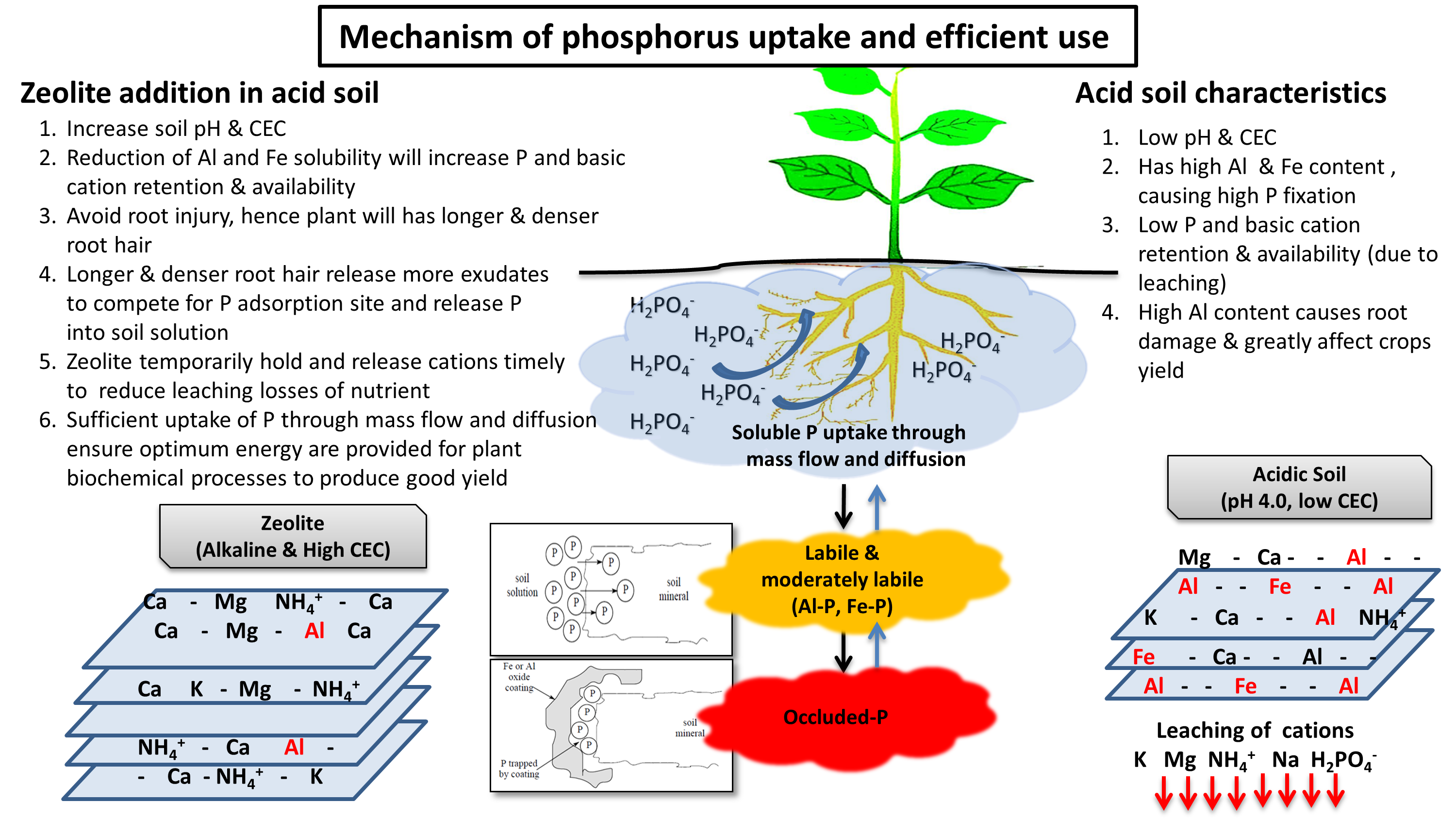

Supplement: S3 Fig — (TIF) [file pone.0204401.s003.tif]
